# Peptides and Amino Acids in Drug Development: Where We Stand and Where We Must Go

**DOI:** 10.3390/biomedicines14030678

**Published:** 2026-03-16

**Authors:** Siva S. Panda

**Affiliations:** 1 Department of Chemistry and Biochemistry, Augusta University, Augusta, GA 30912, USA; sipanda@augusta.edu; Tel.: +1-706-667-4022; 2 Department of Biochemistry and Molecular Biology, Augusta University, Augusta, GA 30912, USA

## 1. Introduction

Peptides are now a key part of modern drug development, transforming the industry at an extraordinary rate [1,2,3]. Once regarded as specialized tools, peptides and amino acids have become essential to pharmaceutical progress [4,5]. The worldwide peptide therapeutics market is expected to exceed $250 billion by 2030, with a compound annual growth rate of over 9%, fueled by advances in metabolic disorders, cancer treatments, and immunotherapy [6]. This growth signals a major change: peptides are not merely replacing small molecules and biologics, but are also expanding the horizons of drug discovery [7].

Peptides and amino acids, nature’s most information-rich molecular building blocks, are no longer confined to the role of biochemical tools or endogenous signaling molecules. They have emerged as a transformative therapeutic modality, bridging the gap between small molecules and biologics. Today, peptide-based drugs represent one of the fastest-growing segments in pharmaceutical innovation, driven by their exceptional target selectivity, structural versatility, and generally favorable safety profiles. These attributes have enabled peptides to tackle previously “undruggable” targets, particularly protein–protein interactions and complex signaling networks, which remain elusive to conventional small molecules [8].

The renaissance of peptide therapeutics is underpinned by decades of technological progress. Advances in solid-phase peptide synthesis, recombinant technologies, macrocyclization chemistry, and rational design strategies have dismantled historical barriers such as poor oral bioavailability, rapid proteolysis, and limited membrane permeability. Concurrently, breakthroughs in delivery systems, including cell-penetrating peptides, nanocarriers, and peptide–drug conjugates, have expanded the clinical reach of peptides beyond injectable formulations toward oral and targeted delivery platforms [9,10]. These innovations have transformed peptides from a theoretical concept into a clinical reality, resulting in nearly 100 approved drugs globally and hundreds more in advanced development stages [11].

The timing of this Special Issue, “Peptides and Amino Acids in Drug Development: Here and Now,” reflects a pivotal moment for the field. Peptide drugs now dominate therapeutic areas such as diabetes, obesity, oncology, and rare diseases, while emerging applications in immunotherapy, infectious disease, and vaccine development underscore their versatility. Market data illustrate this momentum: GLP-1 receptor agonists like semaglutide and tirzepatide have redefined standards of care for metabolic disorders, achieving multi-billion-dollar sales and setting new benchmarks for efficacy and patient compliance. Beyond metabolic disease, peptide–drug conjugates and stapled peptides are reshaping cancer therapy, while peptide-based vaccines are advancing precision immunization strategies.

Unlike conventional reviews, this Special Issue integrates perspectives from chemistry, computational design, and translational delivery science, offering a holistic view of peptide innovation from molecular design to clinical application. By bridging molecular innovation with translational strategies, this collection underscores how peptide science is shaping real-world therapies.

## 2. Why Peptides? Why Now? Relevance in Contemporary Drug Discovery

The renewed interest in peptides for drug development is more than a passing fad; it signals a fundamental shift in how new therapies are developed. Sitting between small molecules and biologics, peptides offer a unique blend of the precision of protein-based drugs and the versatility of chemical synthesis. This combination enables them to tackle some of the biggest challenges in modern medicine, such as achieving high target specificity, reducing systemic toxicity, and influencing complex biological processes, including protein–protein interactions, that are typically out of reach for traditional small molecules.

One of the most compelling advantages of peptides lies in their unmatched selectivity for complex targets. Many high-value therapeutic targets, particularly those involving protein–protein interactions (PPIs), extended binding interfaces, and transient signaling complexes, are difficult for small molecules to engage due to their limited surface coverage [12]. Peptides, with their larger size and conformational flexibility, can complement these interfaces, offering superior specificity and affinity. In oncology, stapled peptides mimicking α-helices have shown promise in disrupting oncogenic PPIs such as p53–MDM2/MDMX, restoring tumor suppressor function in cancers where p53 is inactivated [13]. Clinical candidates like ALRN-6924 exemplify this approach, demonstrating activity in both solid tumors and hematologic malignancies. Similarly, tumor-homing peptides, such as RGD-based sequences, are being incorporated into peptide–drug conjugates (PDCs) for the targeted delivery of cytotoxic agents to integrin-overexpressing tumors, thereby improving efficacy while minimizing systemic toxicity. In immunotherapy, peptides targeting immune checkpoints like PD-1/PD-L1 are emerging as alternatives to monoclonal antibodies, offering lower immunogenicity and improved tissue penetration [14]. Cyclic peptides and peptidomimetics are under development to block PD-1/PD-L1 interactions and enhance T-cell activation in cancer immunotherapy.

Modern peptide design goes far beyond natural amino acid sequences. Chemical innovations now enable the precise tuning of pharmacological properties. Macrocyclization and stapling techniques constrain peptide conformation, enhancing proteolytic stability and cell permeability. For example, stapled GLP-2 analogs have demonstrated improved efficacy in inflammatory bowel disease models. The incorporation of non-canonical residues, such as D-amino acids, N-methylation, and β-amino acids, extends half-life and resists enzymatic degradation [15,16]. Selective backbone modifications have yielded long-acting analogs such as tirzepatide, a dual GIP/GLP-1 agonist for diabetes and obesity. Hybrid constructs that combine peptides with small molecules further expand therapeutic possibilities, merging the selectivity of peptides with the pharmacokinetic advantages of small molecules. GLP-1 receptor agonists such as semaglutide exemplify rational engineering: strategic substitutions and lipid conjugation confer an ultra-long half-life, enabling once-weekly dosing and even oral formulations [17]. Tirzepatide extends this concept by integrating dual agonism for superior glycemic and weight control.

Delivery innovation is equally pivotal to peptide success. Historically limited by poor oral bioavailability and rapid clearance, peptides now benefit from cutting-edge platforms. The approval of oral semaglutide (Rybelsus^®^) marked a milestone, achieved through co-formulation with SNAC (sodium N-[8-(2-hydroxybenzoyl)amino]caprylate), which enhances gastric absorption and protects against enzymatic degradation. Nanocarriers and cell-penetrating peptides (CPPs) enable the intracellular delivery of peptides and nucleic acids, expanding the therapeutic scope into oncology and gene therapy. Peptide–drug conjugates such as Lutetium Lu-177 dotatate (Lutathera^®^) exemplify targeted radionuclide therapy for neuroendocrine tumors, leveraging somatostatin analogs for precision delivery [18]. Emerging formulations for transdermal and intranasal routes, such as those for migraine (CGRP inhibitors) and neurodegenerative disorders, highlight the versatility of peptide delivery systems.

The clinical success of GLP-1 receptor agonists, such as tirzepatide and peptide–drug conjugates, highlights the versatility of peptide-based approaches. These agents are revolutionizing the treatment of metabolic conditions and advancing fields like oncology, immunotherapy, and vaccines. Market forecasts show continued double-digit growth for peptide therapeutics, emphasizing their strategic role in medicine. Overall, peptides do more than just add incremental improvements; they are reshaping drug discovery, combining biological accuracy with chemical adaptability to become essential tools for precision medicine and innovative therapies.

## 3. Persistent Gaps in Knowledge and Practice

Although there has been notable advancement in peptide drug development, several key challenges still hinder wider clinical use and successful translation. These issues are not just technical; they reveal deeper gaps in our understanding of how peptides behave within complex biological systems.

One major hurdle is the unpredictable in vivo performance of peptides [19]. Stability and pharmacokinetics remain highly context-dependent, and chemical modifications that confer proteolysis resistance in one tissue may fail in another due to variations in protease expression, pH, and transport mechanisms. This unpredictability complicates dose optimization and increases the risk of late-stage attrition. For example, while GLP-1 analogs have achieved extended half-lives through lipid conjugation, similar strategies for other peptide classes often yield inconsistent results.

Another persistent challenge is oral bioavailability, which remains the exception rather than the norm. Although oral semaglutide represents a landmark achievement, most peptides exhibit poor membrane permeability and are rapidly degraded in the gastrointestinal tract. Current solutions, such as permeation enhancers, enzyme inhibitors, and co-formulation strategies, offer incremental improvements but lack universal applicability. Developing robust, scalable technologies for oral peptide delivery remains one of the most pressing unmet needs in the field.

Intracellular targeting and endosomal escape pose additional barriers. Peptides designed to modulate intracellular targets must overcome formidable obstacles, including cellular uptake and endosomal entrapment. While cell-penetrating peptides (CPPs) and nanoparticle carriers have shown promise, their clinical translation is hindered by variability in uptake efficiency, potential off-target effects, and immunogenicity concerns. Achieving predictable intracellular delivery without compromising safety remains an ongoing challenge.

Manufacturing complexity and regulatory uncertainty further complicate peptide development. The synthesis of macrocyclic peptides, stapled peptides, and peptide–drug conjugates often involves multi-step processes that are difficult to scale while maintaining purity and reproducibility. Regulatory frameworks for these advanced modalities are still evolving, creating uncertainty around quality standards, stability testing, and comparability assessments. Bridging the gap between laboratory innovation and GMP-compliant manufacturing is essential for accelerating clinical translation.

Ultimately, the absence of strong predictive models drives high attrition rates. Existing computational tools and in vitro assays often fail to capture the complex interactions of peptides within biological systems, including protease activity, immune responses, and transport mechanisms. This highlights the necessity for integrated design approaches that leverage AI-based modeling, high-throughput screening, and physiologically relevant testing platforms to enhance predictability and lower development risks.

## 4. Conceptual and Methodological Advances

The contributions to this Special Issue go beyond mere incremental progress and collectively indicate a significant methodological advance in peptide science. Several common themes emerge, highlighting how innovations in design, synthesis, and delivery are transforming the therapeutic landscape.

A major advancement is the adoption of cyclization as a core design principle. Macrocyclization remains essential in peptide drug development because it limits conformational flexibility, enhances proteolytic stability, improves receptor binding thermodynamics, and often enhances membrane permeability. This issue features various strategies, from head-to-tail cyclization to stapled α-helices, that turn delicate linear sequences into durable, drug-like structures. These methods are especially impactful in cancer treatment, where cyclic peptides targeting integrins and growth factor receptors show improved tumor penetration and selectivity.

Another defining characteristic of modern peptide development is delivery-first thinking. Historically, delivery considerations were addressed late in the development process, but this paradigm is shifting. Contributions in this Special Issue showcase delivery-enabled innovations, including peptide-functionalized nanoparticles for targeted cancer therapy and nucleic acid delivery, cell-penetrating peptides engineered for efficient endosomal escape and intracellular targeting, and oral peptide formulations leveraging permeation enhancers and structural modifications to overcome gastrointestinal degradation. These advances reflect a holistic approach where pharmacology and formulation co-evolve rather than follow a sequential model [20].

The issue also explores expanding the source space, moving beyond conventional peptide origins to include ribosomally synthesized and post-translationally modified peptides, non-ribosomal peptides, and plant-derived cyclotides [21,22,23]. These natural scaffolds offer exceptional stability and bioactivity, inspiring the development of synthetic analogs with improved pharmacokinetics and therapeutic profiles. This diversity broadens the chemical space available for lead discovery and opens new avenues for oral and preventive therapeutics.

Another emerging theme is precision immunomodulation. Peptides are increasingly recognized as fine-tuning agents of immune signaling. Articles in this collection demonstrate how short sequences can recalibrate cytokine networks, modulate checkpoint pathways, and enhance vaccine efficacy without broadly suppressing immunity. This precision is particularly relevant in oncology and autoimmune disorders, where balancing efficacy with immune tolerance remains a central challenge.

In essence, the works featured in this Special Issue illustrate a field in transition, from empirical peptide chemistry to a digitally enabled, delivery-focused, and biologically contextualized discipline. These conceptual and methodological advances are not only expanding the therapeutic potential of peptides, but also redefining the standards for innovation in drug development.

## 5. Translational Threads and Emerging Consensus

The contributions in this Special Issue converge on a clear message: peptide therapeutics have matured from experimental concepts into clinically validated modalities. Several translational themes define the current consensus on what makes peptide-based drugs successful in practice.

Stability is engineered rather than taken for granted. The view of stability as an innate characteristic of peptide sequences has shifted. Metabolic resilience is now deliberately crafted through backbone modifications, cyclization, and the addition of non-natural amino acids. This strategic approach guarantees consistent pharmacokinetics and reduces degradation across diverse biological settings [24].

Efficacy and delivery are optimized together. In the past, peptide development was sequential, focusing first on potency and then on delivery. Today, strategies integrate these factors from the outset. This includes lipid conjugation for prolonged circulation, permeation enhancers for oral delivery, and CPPs for intracellular targeting. Delivery-focused design is now a core part of the process.

Biological context is crucial. Peptide effectiveness depends on factors such as the protease environment, immune system interactions, and tissue-specific transport. Contributors to this Special Issue highlight the importance of context-aware design, leveraging systems biology and pharmacogenomic insights to tailor peptides to specific disease conditions. This transition from a “one-size-fits-all” approach to precision engineering represents a significant advancement in translational science.

Manufacturability and scalability remain key strategic priorities. Moving from research to clinical application depends on reliable, GMP-compliant manufacturing processes. Progress in solid-phase synthesis, automation, and green chemistry is reducing complexity and environmental impact. Experts emphasize the need for early coordination between discovery chemistry and process development to prevent bottlenecks later in development.

Digital integration accelerates translation. Machine learning and AI-driven models are now essential, playing a key role in peptide drug development. These predictive algorithms assist with sequence optimization, stability assessment, and delivery system design, reducing reliance on trial-and-error approaches and shortening the path to clinical trials.

## 6. Broader Context and Field Trajectory

Peptide therapeutics have progressed from niche applications to a cornerstone of modern drug discovery. Initially limited to hormone replacement and rare diseases, peptides now address metabolic disorders, oncology, infectious diseases, immunology, and vaccines. Rather than replacing small molecules or biologics, peptides complement both, expanding the design space for precision medicine and enabling solutions for previously intractable targets. Their integration into combination therapies, targeted delivery systems, and vaccine platforms underscores their versatility and enduring relevance in the global pharmaceutical landscape.

Recent advancements have sparked renewed interest in naturally occurring peptides with remarkable biological potency, as demonstrated by amatoxins such as α-amanitin, the toxin found in the death cap mushroom. Long celebrated for their selective inhibition of RNA polymerase II, these bicyclic peptides underscore both the potential and the challenges of peptide drug development: they offer extreme target specificity but are associated with dose-limiting toxicity and delivery challenges. Natural cyclic peptides stand out for their unique potential for activity. However, the synthesis of cyclic peptides remains a significant challenge for researchers due to their complex structural characteristics. Notably, Perrin’s research group achieved the first total synthesis of α-amanitin [25].

Modern strategies emphasize precision delivery over free peptide administration, particularly through antibody–drug conjugates (ADCs) and peptide-guided targeting systems, enabling controlled intracellular delivery while minimizing systemic exposure. Preclinical studies show α-amanitin-based ADCs exert potent antitumor activity across pancreatic, colorectal, HER2-low breast, prostate, and multiple myeloma models, with favorable tolerability in mice and non-human primates. Tumors harboring the hemizygous loss of POLR2A, often co-deleted with TP53, exhibit heightened sensitivity to amanitin conjugates, revealing a unique therapeutic vulnerability independent of p53 status [26]. Beyond antibodies, innovative carriers, such as FGF2-based conjugates and small-molecule drug conjugates (SMDCs), aim to improve tissue penetration and pharmacokinetics, thereby addressing limitations in solid tumor delivery. These efforts demonstrate how rational conjugation, linker chemistry, and receptor-mediated uptake can transform historically toxic natural peptides into viable therapeutic payloads.

Recent reviews reinforce the transformative potential of peptide therapeutics across oncology, infectious diseases, and chronic conditions. Sharma et al. highlight innovations such as enzyme-instructed self-assembly, azurin-derived peptides, and peptide-based vaccines, alongside delivery breakthroughs, such as mucus-penetrating peptides for cystic fibrosis and nanocarrier-enabled formulations for glioblastoma. Chemical strategies, cyclization, and PEGylation continue to enhance stability and bioavailability [27].

Complementing these insights, Fetse et al. [28] describe a paradigm shift toward precision engineering and digital innovation. Platforms such as phage and mRNA display, combined with AI-driven design, accelerate the discovery of high-affinity macrocyclic peptides and non-natural variants. Chemical modifications, cyclization, D-amino acid substitution, peptoid formation, and N-methylation are redefining stability and pharmacokinetics, while delivery breakthroughs, such as oral semaglutide and nanoparticle systems, expand clinical accessibility. These trends underscore the convergence of chemistry, computation, and formulation science, positioning peptides as central to next-generation therapeutics.

Han et al. [29] highlight a dual trajectory: refining conventional peptide drugs and pioneering circRNA-encoded polypeptides. Traditional peptides (e.g., GLP-1 analogs, immune-modulatory sequences) maintain efficacy in metabolic disorders, cancer, and autoimmune diseases, aided by nanocarrier delivery and chemical modification. Simultaneously, circRNAs emerge as a novel source of bioactive peptides via IRES-driven and m6A-mediated translation, modulating glycolysis, lipid metabolism, and immune signaling, offering unprecedented opportunities for precision oncology and immunotherapy. While challenges in stability, immunogenicity, and large-scale synthesis persist, integrating circRNA biology with peptide engineering aligns with the Special Issue’s focus on design–delivery synergy and translational innovation.

**Figure 1:**
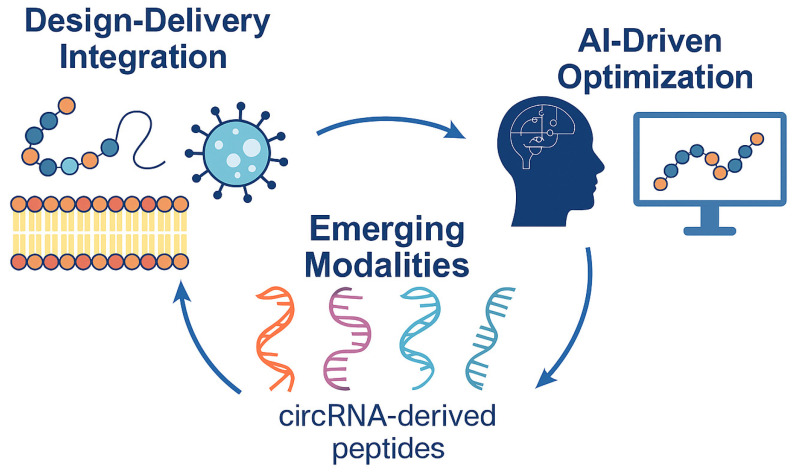
Emerging frontiers in peptide drug development.

Figure 1 highlights three transformative themes shaping next-generation peptide therapeutics: Design–Delivery Integration (co-optimization of molecular structure and formulation for stability and targeted delivery), AI-Driven Optimization (computational modeling and machine learning for rapid peptide design and prediction), and Emerging Modalities such as circRNA-derived peptides that expand the chemical and biological space for innovative therapies.

## 7. Future Directions

The future in peptide drug development is poised to be characterized by the convergence of multiple disciplines, specifically molecular design, delivery science, and digital technologies, to forge therapies that are precise, scalable, and environmentally friendly. Several strategic priorities and emerging opportunities merit attention.

Design and delivery should be integrated as the standard practice. Future development pipelines need to shift from the traditional linear approach of “design first, then deliver.” Instead, molecular structure and formulation should be optimized simultaneously from the beginning to ensure stability, target specific tissues, and enable patient-friendly administration methods. The success of oral peptide therapeutics like semaglutide highlights the powerful potential of adopting a delivery-first approach.

Mechanism-guided engineering will act as a catalyst for more rational design. Advances in protease mapping, structural biology, and computational modeling will facilitate customized backbone modifications and amino acid substitutions suited to specific disease environments. This approach reduces reliance on trial-and-error methods and enhances peptides’ ability to operate effectively within intricate biological systems.

Translational acceleration is critically important. To effectively move from discovery to market, it is vital to develop harmonized Chemistry, Manufacturing, and Controls (CMC) workflows, scalable synthetic methods, and well-defined regulatory frameworks for advanced modalities like peptide–drug conjugates and nano-enabled systems. Aligning innovative efforts early with industrial feasibility helps lower late-stage attrition rates [30].

Peptide drug development is undergoing a significant transformation due to digital technologies and sustainability principles. AI is playing a crucial role by shifting from manual screening to predictive and generative models, thereby enhancing the accuracy of forecasting bioactivity, toxicity, and target binding. Techniques like GANs, VAEs, and large language models are enabling the creation of new peptides. AI platforms, such as AlphaFold and RoseTTAFold, improve the design of protein–peptide interactions and support pharmacokinetic modeling and toxicity assessments [31]. The focus on computational and AI-driven design is reducing reliance on traditional trial-and-error methods, accelerating the development of multifunctional peptides, and facilitating rapid iteration toward clinical candidates.

## 8. Conclusions

The overarching message of this Special Issue is clear: peptides and amino acids have moved from supporting roles in drug discovery to central pillars of therapeutic innovation. The contributions presented here, spanning macrocyclic scaffolds, multifunctional conjugates, immunomodulatory peptides, and advanced delivery systems, demonstrate both conceptual depth and translational impact.

Looking ahead, success will depend on three key factors: integrating design and delivery, mechanism-guided engineering, and accelerating digital processes. New opportunities, such as AI-powered peptide design, peptides from circRNA, and green manufacturing, are poised to revolutionize the therapeutic field and reshape precision medicine.

Will the next blockbuster drug be a peptide? The evidence suggests it will, but only if we embrace design–delivery synergy and digital innovation. Achieving this vision will require cross-disciplinary partnerships spanning chemistry, biology, computation, and clinical science. By adopting these principles, the field can overcome persistent challenges in stability, bioavailability, and scalability, ushering in a new era of peptide-based medicines with global reach.

I hope this Special Issue not only informs readers about the current state of peptide science but also inspires new ideas, fosters collaborations, and catalyzes research that will define the next generation of peptide therapeutics.
